# Preventing malaria in pregnancy through community-directed interventions: evidence from Akwa Ibom State, Nigeria

**DOI:** 10.1186/1475-2875-10-227

**Published:** 2011-08-05

**Authors:** Joseph C Okeibunor, Bright C Orji, William Brieger, Gbenga Ishola, Emmanuel 'Dipo Otolorin, Barbara Rawlins, Enobong U Ndekhedehe, Nkechi Onyeneho, Günther Fink

**Affiliations:** 1 Department of Sociology/Anthropology, University of Nigeria, Nsukka, Nigeria; 2 Jhpiego, 1615 Thames Street, Baltimore, MD 21231-3492, USA; 3 Harvard School of Public Health; 665 Huntington Avenue, Boston, MA, USA; 4 Johns Hopkins Bloomberg School of Public Health, 615 N. Wolfe Street, Baltimore, MD, 21205, USA; 5 Community Partners for Development, Akwa Ibom, Nigeria

## Abstract

**Background:**

Despite massive anti-malaria campaigns across the subcontinent, effective access to intermittent preventive treatment (IPTp) and insecticide-treated nets (ITNs) among pregnant women remain low in large parts of sub-Saharan Africa. The slow uptake of malaria prevention products appears to reflect lack of knowledge and resistance to behavioural change, as well as poor access to resources, and limited support of programmes by local communities and authorities.

**Methods:**

A recent community-based programme in Akwa Ibom State, Nigeria, is analysed to determine the degree to which community-directed interventions can improve access to malaria prevention in pregnancy. Six local government areas in Southern Nigeria were selected for a malaria in pregnancy prevention intervention. Three of these local government areas were selected for a complementary community-directed intervention (CDI) programme. Under the CDI programme, volunteer community-directed distributors (CDDs) were appointed by each village and kindred in the treatment areas and trained to deliver ITNs and IPTp drugs as well as basic counseling services to pregnant women.

**Findings:**

Relative to women in the control area, an additional 7.4 percent of women slept under a net during pregnancy in the treatment areas (95% CI [0.035, 0.115], p-value < 0.01), and an additional 8.5 percent of women slept under an ITN after delivery and prior to the interview (95% CI [0.045, 0.122], p-value < 0.001). The effects of the CDI programme were largest for IPTp adherence, increasing the fraction of pregnant women taking at least two SP doses during pregnancy by 35.3 percentage points [95% CI: 0.280, 0.425], p-value < 0.001) relative to the control group. No effects on antenatal care attendance were found.

**Conclusion:**

The presented results suggest that the inclusion of community-based programmes can substantially increase effective access to malaria prevention, and also increase access to formal health care access in general, and antenatal care attendance in particular in combination with supply side interventions. Given the relatively modest financial commitments they require, community-directed programmes appear to be a cost-effective way to improve malaria prevention; the participatory approach underlying CDI programmes also promises to strengthen ties between the formal health sector and local communities.

## Background

Despite a massive increase in private and public efforts over the last years, malaria remains one of the most salient global health concerns. According to the latest estimates, 250 million cases of malaria are recorded each year, with an estimated associated annual toll of 781,000 deaths [1]. The majority of the burden of disease caused by malaria is borne by the populations living in the highly endemic areas of sub-Saharan Africa. Within these areas, the populations at highest risk are pregnant women and infants[2]. Malaria infection in pregnancy is a major risk factor for maternal and child health, and substantially increases the risk of miscarriage, stillbirth and low birthweight [3,4]. In sub-Saharan Africa alone, approximately 25 million pregnant women are at risk of *Plasmodium falciparum *infection every year. Approximately one in four women show evidence of placental infection at the time of delivery, with a large fraction of infection remaining undetected and untreated [5]. The health consequences of malaria infection during pregnancy are large: malaria-induced low birthweight is estimated to account for up to 360,000 infant deaths every year[6]; overall, 11.4% of neonatal deaths and 5.7% of infant deaths in malaria-endemic areas of Africa are estimated to be caused by malaria in pregnancy [7,8].

In order to prevent malaria in pregnancy, current WHO guidelines recommend a multi-pronged approach including both preventive and curative measures [9,10]. The Focused Antenatal Care approach recommends the use of insecticide-treated nets (ITN) early in pregnancy and a minimum of two treatment doses of sulphadoxine-pyrimethamine (SP) as intermittent preventive treatment in pregnancy (IPTp) [10]. A meta-analysis of intervention trials suggests that successful prevention of infection reduces the risk of severe maternal anaemia by 38%, low birthweight by 43% and perinatal mortality by 27% among paucigravidae [10]. Garner *et al *estimate that effective prevention of malaria with chloroquine prophylaxis or IPTp reduces the risk of low birthweight by as much as 43% [11].

Despite these recommendations, effective access to malaria prevention in pregnancy remains limited in Nigeria. According to the 2008 Nigeria Demographic and Health Survey, only 11.8% of pregnant women slept under an ITN, and only 6.5% of pregnant women had taken the recommended two doses of SP during pregnancy [12]. Accordingly, the prevalence of malaria in pregnancy remains high, with recent estimates suggesting prevalence rates of close to 50% in the second and third trimesters [13].

In an attempt to improve effective access to malaria prevention in pregnancy, Jhpiego - an international non-profit health organization affiliated with The Johns Hopkins University -launched the project evaluated in this paper with funding support from ExxonMobil Foundation in 2008. Jhpiego focuses on strategies to help countries care for themselves by training competent health care workers, strengthening health systems and improving delivery of care [14]. The primary objectives of the intervention were to increase net use as well as the uptake of IPTp by pregnant women. The project also aimed at increasing ANC (antenatal care) center attendance and at quality improvement of health services. To reach these objectives, a two-pronged approach was adopted, combining the provision of additional resources and training of health center staff with community-directed interventions as described in further detail below.

The project was implemented in close collaboration with the Ministry of Health. The main partners within the Ministry were members of the RBM Unit (Malaria Control Programme), the Reproductive Health Programme, the Onchocerciasis Control Programme unit, the Monitoring and Evaluation unit, as well as the State Pharmacy stores. These stakeholders became the state Malaria/CDI core training team, who trained local core teams and supported monitoring and evaluation efforts.

The focus on community directed interventions in the design of the project was driven by the large theoretical and empirical literature highlighting the importance of community involvement in the delivery of health services in general, and preventive health measures in particular [15-22]. While the involvement of community workers does not necessarily mediate socioeconomic differences within communities [23], the overall health improvements achievable through community based interventions appear large [16]. The achievable improvements appear particularly large in cases where communities actively make program decisions [24], and where community programme rollout is further delegated to local families and kinships [25].

## Methods

### Study design

The study used a pre-post parallel group design, with group assignment implemented at the local government area level (LGA). Each LGA comprises an average population of approximately 130,000 individuals and an estimated average number of 6500 pregnancies per year. Three LGAs (Eket, Esit Ekit, Onna) were assigned to the treatment group, and three LGAs (Ikot Abasi, Mbo, Mkpat Enin) were assigned to the control group. The assignment of areas to treatment and control was not random, but rather determined prior to the beginning of the study with the objective to guarantee balanced samples with respect to health facility infrastructure across the two study arms. Independent random samples of women with pregnancies over the six-month period preceding the survey were interviewed pre- and post-intervention to measure changes in effective access to malaria prevention in pregnancy.

### Participants

**Figure 1:**
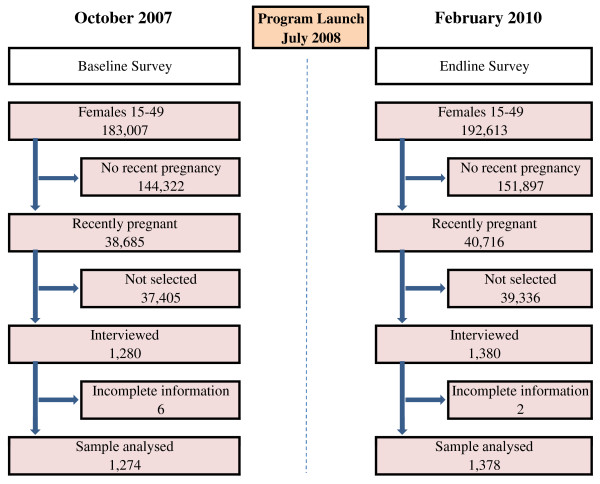
**Sampling and time line**.

Target population of the programme was all pregnant women residing in the six programme LGAs. As Figure 1 illustrates, 1,280 women aged 15-49 with recent pregnancies in the study areas were randomly selected for an interview at baseline. A second, independent sample of 1,380 women was randomly selected for a follow-up interview in February 2010. Target populations of both surveys were women who had given birth within six months prior to the survey.

### Study settings

The study was located in the Eket Senatorial Zone in Southern Nigeria. Eket Senatorial Zone is one of three senatorial zones within Akwa Ibom State, and the principal area of operation of ExxonMobil in the region. Akwa Ibom State covers a landmass of approximately 8,000 square kilometers and is currently home to an estimated population of 3.9 million [26]. The discovery and extraction of crude oil in the area has led to massive in-migration over the last decades, resulting in a rapidly growing ethnically diverse population. Local climate is tropical, with a dry season between November and March and a wet season between April and October. The average temperature ranges from 23°C - 31°C, providing an ideal climate for malaria transmission throughout the year, and placing Akwa Ibom State among the areas with the highest malaria transmission in the whole region [27]. Even though only 2.5% of Nigeria's population lives in the state, Akwa Ibom accounts for over 11% of malaria-linked maternal mortality and 12-30% of under-5 mortality due to malaria in the country [28].

**Figure 2:**
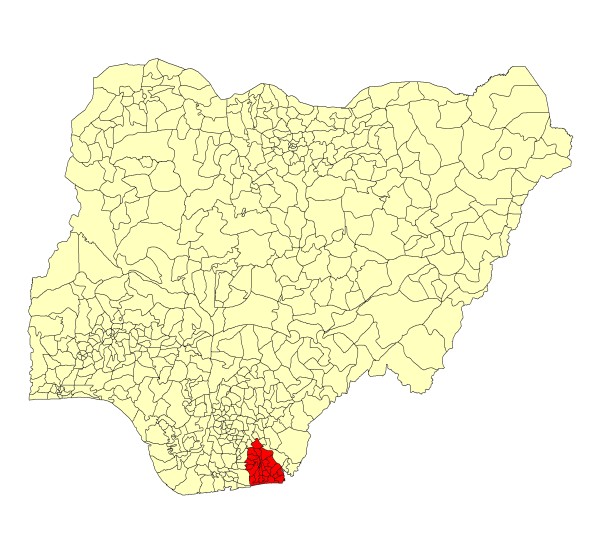
**Nigeria and Akwa Ibom State**.

**Figure 3:**
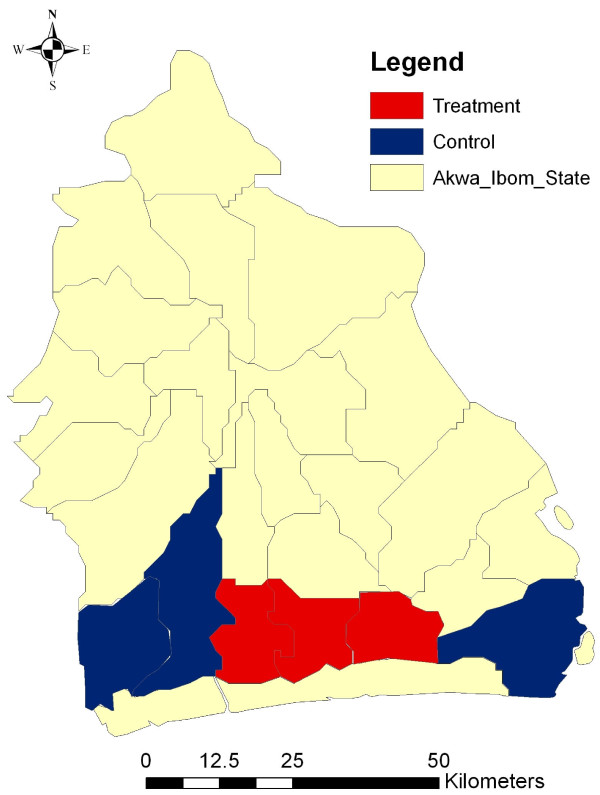
**Target areas within Akwa Ibom State**.

As illustrated in Figures 2 and 3, the target area of the study covers six LGAs within the Eket Senatorial zone along the coastal belt of Akwa Ibom State: Eket, Esit Eket, Ikot Abasi, Mbo, Mkpat Enin and Onna. Each of the six local government areas is served by at least one state government hospital, and all LGAs have at least four additional health clinics or health posts. Overall, there are 20 Local Government Primary Health Care facilities in the treatment area, and 19 health facilities in the control area.

### Interventions

In order to minimize equity concerns, increased resource and training support was provided to both treatment and control areas. All public health clinics in the programme area were provided with drugs, functional equipment and other supplies such as tracking sheets to enhance their functionality. Health workers received additional training focusing on the delivery of ANC services at public health clinics in general, and on improving malaria in pregnancy (MIP) services in particular. MIP performance standards were developed to improve service delivery and workers from health facilities in the control and intervention arms were trained to achieve these standards.

In addition to these common interventions, a CDI programme was implemented in the three treatment LGAs. The main objective of the CDI was to select and train local delivery agents (CDDs) to support malaria prevention efforts. To ensure local support, the selection of CDDs was delegated to each kindred within a given community. Similar to the kindred definition described by Katabarwa *et al *for the Ugandan context [29], "kindreds" are extended family units or clans with a common ancestry, commonly referred to as "ekpuk" by the local Ibibios, and as "Umunna" in neighbouring areas of Nigeria [30,31]. Typically, Ibibio villages held about five hundred people, and were divided into physically distinct divisions dominated by separate patrilineages. Priority in the selection of CDDs was given to women from each local kindred with prior childbearing experience in order to minimize communication barriers between pregnant women and the CDDs. Overall, 700 volunteer community-directed CDDs were trained from more than 450 kindred groups. With an estimated 86,000 women between 15 and 49, this implies that approximately one CDD was trained for every 120 women of childbearing age in the treatment areas. With a estimated general fertility rate of 194 per 1000 women of childbearing age [12], this implies that each CDD covered on average 23 births per calendar year.

CDDs were trained to deliver ITNs and SP (IPTp1 and IPTp2) to pregnant women in the treatment arm communities, and also to provide basic health counseling services. To make sure the CDI programme would not undermine usage of the existing health system, CDDs were instructed to refer pregnant women to health facilities for additional ANC services. Conditional on successful completion of the training, CDDs were equipped with counseling cards, IPTp drugs and ITNs, as well as village register, referral forms and monthly tally sheets to report service statistics. All training and supervision of the CDDs was conducted by staff of the nearest health facility within the treatment area to ensure full collaboration between health facility employees and CDDs.

### Time line

Baseline surveys were conducted from October 1^st ^to October 15^th ^2007. The rollout of the interventions started in July 2008; interventions continued throughout 2010. The follow-up survey interviews were conducted between February 11^th ^and February 26^th ^2010.

### Outcomes

The primary objective of the intervention was to increase effective access to malaria prevention among pregnant women; specific targets were increasing ITN use, and increasing access to IPTp in the form of two doses of SP during pregnancy. The effectiveness of the intervention is evaluated through five health access indicators: the probability of a woman (1) reporting to access ANC services during her pregnancy at least once; (2) reporting to have slept under a ITN during pregnancy; (3) reporting to have slept under a ITN the night before the interview; (4) reporting having taken any malaria prevention drugs; (5) reporting having taken at last two doses of SP (each dose of SP consists of three tablets).

### Sample size

A randomly-selected sample of 1,280 women were interviewed at baseline. Data analysis was restricted to women with complete information, which results in a final baseline sample of 1,274 respondents. An independent random sample of 1,380 women were interviewed at endline. Two women had missing information on at least one key variable, resulting in an endline sample of 1378 observations. The sample size for the household surveys was calculated in order to be able to detect a 10 percentage point increase in utilization of government ANC services from a baseline of 25% of pregnant women reporting at least one ANC visit at a government health facility.

### Statistical methods

Primary endpoints of the study were the fraction of pregnant women sleeping under ITNs, as well as the fraction of women taking the recommended two doses of SP. Secondary outcomes included ANC visits, ITN use post-pregnancy, as well as other malaria prevention efforts.

Unconditional pre-post group mean differences are presented as a first step, before showing multivariate regression results. In order to facilitate coefficient interpretation, heterogeneity-adjusted linear probability models of the following functional form are estimated:(1)

*y *is the outcome of interest for woman *i *in local government area *j *and period *t*, and *POST *is a binary indicator, which equals 0 for baseline (PRE) observations and 1 for end line (POST) observations. The *POST *term captures the average improvements in both groups, while the POST * *CDI *interaction term captures differences in changes across the two groups, and thus represents the treatment effects of interest. *X *is a vector of control variables, which includes respondent's age, education, marital status, ethnicity, religion, occupation and household wealth. In order to capture time-invariant characteristics specific to the six local government areas, LGA fixed effects *δ_j _*are included in the empirical models. Given that all dependent variables are binary, Huber-White standard errors are applied to adjust for the non-normal distribution of the error terms. To ensure correct causal inference in the presence of spatial correlation within the treatment areas, all standard errors are clustered at the LGA level.

In order to ascertain that the main results are not driven by differences in access at baseline, a series of small-sample estimates representing pair-wise comparisons of the two most similar LGAs for each outcome of interest are also reported. All empirical analysis was conducted using the Stata^© ^10 statistical software package.

### Ethical approval

The study was registered with the Federal Ministry of Health in Abuja. Prior to the rollout of the programme, a consensus-building meeting was held with senior members of the State Ministry of Health to agree on the services CDDs would provide. In addition, health workers engaged with community stakeholders in local meetings to seek their approval, sensitize them towards the importance of their role in promoting maternal health, encourage shared learning and create a supportive environment for the programme.

Ethical approval was obtained through the Committee on Human Research at Johns Hopkins Bloomberg School of Public Health, Baltimore, Maryland, USA; and the National Malaria Control Programme (NMCP), Abuja approved the study protocol for implementation. A formal MOU was set up between Jhpiego and local as well as State authorities. Informed and written consent was obtained from all persons who voluntarily agreed to be interviewed.

## Results

### Population characteristics

Table 1 summarizes the socio-demographic characteristics of the sample population. The mean age of respondents is 25.5 years, with a majority of women in both areas belonging to the Ibibio ethnicity. The treatment areas do not only have a slightly higher fraction of women belonging to the Ibibio ethnicity, but also differ with respect to marital status and schooling. A higher fraction of women are single in the control areas, and on average educational attainment is also higher in the treatment areas. Following the methodology proposed in Filmer and Pritchett [32], principal component analysis is used to compute an asset index, and divide households into five asset quintiles. Consistent with the results on educational attainment, households in the treatment area appear on average slightly wealthier; the null of equal wealth levels across group could not be rejected at a 95% confidence interval.

**Table 1:** Respondent Demographic Characteristics

	Treatment				Control			
	*Baseline*		*Endline*		*Baseline*		*Endline*	
	n = 711		n = 751		n = 563		n = 627	
Age, *mean (SD)*	25.5	(5.96)	26.1	(10.63)	25.1	(6.24)	25.1	(5.97)
Ethnicity Ibibio, *N (%)*	617	(33.9)	705	(24.4)	347	(48.7)	399	(48.1)
Ethnicity Anang, *N (%)*	33	(21.1)	19	(15.7)	29	(22.1)	15	(15.3)
Ethnicity Aran, *N (%)*	20	(16.5)	18	(15.3)	171	(46.0)	199	(46.6)
Catholic, *N (%)*	25	(18.4)	40	(22.4)	76	(34.1)	41	(24.7)
Protestant, *N (%)*	344	(50.0)	373	(50.0)	255	(49.8)	311	(50.0)
Single, *N (%)*	85	(32.5)	136	(38.5)	123	(41.3)	135	(41.1)
Married, *N (%)*	621	(33.3)	611	(39.1)	424	(43.4)	486	(41.8)
No schooling, *N (%)*	25	(18.4)	22	(16.9)	45	(27.1)	43	(25.3)
Primary Schooling, *N (%)*	262	(48.3)	217	(45.3)	222	(48.9)	201	(46.7)
Secondary schooling, *N (%)*	356	(50.0)	454	(49.0)	267	(50.0)	345	(49.8)
Tertiary schooling, *N (%)*	67	(29.2)	59	(26.9)	30	(22.4)	37	(23.6)
Working, *N (%)*	368	(50.0)	386	(50.0)	289	(50.0)	293	(49.9)
Wealth quintile, *mean (SD)*	3.0	(1.5)	3.4	(1.4)	2.6	(1.4)	2.9	(1.3)

SD Standard deviation.

There were also some baseline differences between women in the treatment and control groups with respect to effective access to health services. As Table 2 shows, women in the treatment areas were more likely to have visited an ANC center during pregnancy at baseline, and also to have received a tetanus vaccine and iron supplements. Women in the treatment group were also more likely to sleep under an ITN and having taken two doses of SP prior to the intervention.

**Table 2:** Effective access to health services at baseline

	Treatment		Control	
	N = 711		N = 563	
*Health Access, N (%)*				
Any ANC visit	489	(68.8)	283	(50.0)
Received Tetanus vaccine	388	(54.6)	244	(43.3)
Received iron supplements	565	(79.5)	375	(66.6)
				
*Malaria Prevention, N (%)*				
Took malaria prevention drug	480	(67.5)	284	(50.4)
Took at least two doses Fansidar	66	(9.3)	35	(6.2)
Slept under a net while pregnant	194	(27.3)	103	(18.3)
Slept under net last night	128	(18.0)	48	(8.5)

### Programme impact

Table 3 compares the unconditional group means for the outcome variables and the treatment and control areas, pre- and post-intervention. As the table shows, all five outcome measures improved in both the treatment and control areas over the observation period. The most substantial improvements were observed in terms of ANC center visits and IPTp. In the control group, the fraction of women taking the proper two doses of IPTp increased from 6 percentage points to 27 percentage points; in the treatment group, the corresponding percentage of women increased from 9 percentage points to 66 percentage points. The fraction of women visiting an ANC center at least once increased from 50 to 72 percentage points, and from 69 to 0.90 percentage points in the control and treatment areas, respectively. The improvements for ITN use were more moderate, with increases of less than 5 percentage points in the control areas, and of about 10 percentage points in the treatment areas.

**Table 3:** Pre-post group mean comparison

	Control				Treatment			
	Pre	Post	Post-Pre	p-value	Pre	Post	Change	**p-value**^**a**^
At least one ANC visit	0.50	0.72	0.22	0.00	0.69	0.90	0.21	0.00
Slept under net during pregnancy	0.18	0.21	0.03	0.07	0.27	0.38	0.11	0.14
Slept under net before interview	0.08	0.10	0.02	0.51	0.18	0.28	0.10	0.12
Took malaria prevention drug	0.50	0.67	0.17	0.06	0.68	0.87	0.20	0.00
Took at least 2 SP doses	0.06	0.27	0.21	0.01	0.09	0.66	0.57	0.00

Standard errors underlying the reported p-values are clustered at the LGA level.

Table 4 shows the main CDI treatment effects as estimated in the empirical model described in equation (1). The first row of Table 4 (POST) shows the increase in the five outcome variables achieved in both groups, while the interaction term coefficient (POST*CDI) captures the additional improvements achieved in the CDI areas conditional on the full set of covariates described in Table 1.

**Table 4:** Multivariate regression results

	At least one ANC visit	Slept under net during pregnancy	Slept under net before interview	Took malaria prevention drug	Took at least 2 SP doses
Post	0.177	0.029	0.013	0.139	0.203
	(0.095 - 0.259)	(0.018 - 0.041)	(-0.008 - 0.034)	(0.028 - 0.251)	(0.131 - 0.274)
Post*CDI	-0.016	0.074	0.085	0.039	0.353
	(-0.107 - 0.074)	(0.035 - 0.113)	(0.048 - 0.122)	(-0.081 - 0.159)	(0.280 - 0.425)

*Notes*: 95% confidence intervals in parentheses. Results reflect coefficients from a heteroskedasticity-adjusted linear probability model. Numbers in parentheses are standard errors clustered at the local government area (LGA) level. Multivariate controls include age, ethnicity, religion, marital status, respondent's educational attainment, occupational status and household wealth as measured by a principal component-based asset index. Based on a sample of 2652 observations.

Conditional on all observable covariates, the CDI programme had the largest effect on IPTp, increasing the fraction of women taking at least two doses of SP by a total of 55 percentage points, more than twice the increase experienced in the control group. The CDI treatment also increased the likelihood of women reporting to sleep under an ITN both during and after pregnancy; relative to the control group, the fraction of women reporting to sleep under an ITN during pregnancy and the night before the interview increased by an additional 7.4 and 8.5 percentage points, respectively.

### Robustness checks

The results presented in section 3.2 suggest that the addition of CDI programmes was associated with substantial increases in the effective access to malaria prevention among pregnant women. The degree to which these additional increases reflect the causal effect of CDI programmes, however, is contingent on the validity of the common trend assumption underlying the empirical model used. While the basic group mean difference approach in this paper does not require identical baseline characteristics for accurate causal inference, the observed differences in independent and outcome variables at baseline could, at least in theory, affect outcome trajectories over the programme period. Wealthier and more educated women could be more responsive to the CDI programme, or, alternatively, already have more access, and thus have less room or need for improvement. To investigate these concerns, two separate robustness check were conducted. As a first robustness check, analysis was restricted to the four LGAs most comparable in terms of their baseline socioeconomic characteristics. In the programme area, the most comparable LGAs were the neighboring areas of Esit Eket and Onna in the treatment, and Mbo and Mkpat Enin in the control areas, respectively. If it was true that baseline socioeconomic characteristics drive the results, smaller estimated effects should be observed in this sub-sample. As the results in the top panel of Table 5 (Panel A) show, the coefficients do indeed change in the expected direction. However, the magnitude of these changes is small, so that the observed coefficients are not statistically different from the full sample coefficients reported in Table 5 at standard confidence levels. To confirm that the estimated results are not driven by differences in effective access at baseline, a second robustness check was implemented where the empirical model is restricted to pairwise matched treatment and control groups. Given the pronounced differences in baseline access across LGAs and outcome variables, the most comparable pair of treatment and control LGAs for each outcome of interest was selected. For IPTp, Onna with baseline access to IPTp of 8.3% was compared to Mkpat Enin with baseline IPTp access of 7.3%. For ITN use, women in Eket were matched to women in Mbo (baseline 17.1% vs. 17.5%). For ANC visits, women in Ikot Abasi were compared with women in Onna. The results reported in Panel B of Table 5 suggest that, if anything, the targeting of the CDI interventions to the more developed central LGAs may lead to an under- rather than an over-estimation of true programme effects. Compared with the main results presented in Table 4, all coefficients display increased magnitude and significance, suggesting that the observed access differences at baseline suppress rather than inflate the average impact of the CDI programme.

**Table 5:** Robustness checks

	At least one ANC visit	Slept under net during pregnancy	Slept under net before interview	Took malaria prevention drug	Took at least 2 SP doses
*Panel A: Bordering LGAs only*					
CDI effect	-0.047	0.051	0.090	-0.002	0.328
	(-0.188 - 0.093)	(-0.007 - 0.110)	(0.049 - 0.130)	(-0.168 - 0.164)	(0.217 - 0.440)
Observations	1732	1732	1732	1732	1732
R-squared	0.24	0.08	0.09	0.15	0.32
					
*Panel B: Pairwise Matching*					
CDI Effect	0.086	0.090	0.048	0.166	0.401
	(0.079 - 0.094)	(0.068 - 0.113)	(0.017 - 0.079)	(0.141 - 0.192)	(0.389 - 0.413)
Treatment LGA	Onna	Eket	Eket	Ikot Abasi	Ikot Abasi
Control LGA	Mbo	Mbo	Mbo	Onna	Onna
Observations	934	914	914	940	940
R-squared	0.184	0.048	0.041	0.122	0.319

*Notes*: 95% confidence intervals in parentheses. Results reflect coefficients from a heteroskedasticity-adjusted linear probability model. Numbers in parentheses are standard errors clustered at the local government area (LGA) level. Multivariate controls include age, ethnicity, religion, marital status, respondent's educational attainment, occupational status and household wealth as measured by a principal component-based asset index. Based on a sample of 2652 observations.

### Interpretation

The results presented suggest that the addition of the CDI programme leads to large and statistically significant increases in effective access to malaria prevention in pregnancy. The CDI effects appear largest for IPTp, which are relatively easily available and can be administered directly at pregnant women's homes in the presence of CDDs. While ITN uptake was also increased through the CDI programme, progress was more limited compared to IPTp. This difference may be partly explained by limited availability of ITNs in some study areas; one may also view it as evidence for the slow pace at which local behaviour can be changed even if health goods are freely provided, and even if free distribution campaigns are supported by educational programmes.

From a policy perspective, two things are important to stress: first, the additional costs generated by the CDI programme appear rather small when compared to larger health campaigns. The total first-year programme cost (exclusive of research and research management cost) was US$ 60,100 for an area with a total population of over 750,000 individuals. Given that a majority of this cost was due to training, costs were substantially lower in subsequent years, with a total annual cost of US$ 27,000 or about 25 cents per woman of age 15-49. Compared to larger IRS spraying or ITN campaigns, this cost appears small, and should easily be absorbable in larger national anti-malaria campaigns.

The second point of particular note is that CDI programmes do not appear to crowd out formal health care visits. A fear frequently voiced at the beginning of the programme was that home-based delivery of health services through CDDs might reduce attendance to antenatal health clinics. This clearly was not the case; in fact, one of the most positive changes induced by the programme was the pronounced increase in ANC visits across all programme areas. The likelihood of a pregnant woman visiting an ANC center at least once during her pregnancy increased from 0.50 to 0.72 in the control, and from 0.69 to 0.90 in the treatment area, which is clearly not in line with the notion that community involvement must always reduce ANC center visits.

## Conclusion

Despite massively increased resources toward the prevention and treatment of malaria, effective access to malaria prevention remains low in large parts of sub-Saharan Africa today. With limited health system capacities, and lack of knowledge and resource demand at the household level, national malaria campaigns struggle to deliver resources to large parts of the often most exposed populations. The results of this study suggest that CDI programmes may offer a simple and effective way to increase uptake of malaria prevention. These findings appear consistent with the results from a recent multi-country study conducted by the WHO which found that, conditional on sufficient training and support, community implementers can effectively deliver essential health care to women of reproductive age, and support the delivery of IPTp and ITN services as well as counseling to communities [33].

While CDI programmes may be beneficial to a region or country even in the absence of malaria prevention resources - by strengthening the ties between the formal health sector and local communities, for example - major improvements in malaria prevention are clearly only possible if sufficient resources and supplies are available at health facilities, which is still not the case in many parts of the Sub-Saharan continent. In the context of malaria, CDI programmes should thus not try to substitute for other anti-malaria programmes, but rather to complement and support larger programmes by increasing the effective access to, and use of, distributed resources. Given the slow adoption of nets and preventive treatment in many areas with massive distribution campaigns, CDI strategies definitely appear a highly attractive option to improve the efficiency of these national and regional efforts.

More generally, the training and involvement of community volunteers through health facility staff has the potential to strengthen ties with the formal health sector and to increase its reach into often underserved rural or marginalized communities. In the case of Akwa Ibom State, the CDI programme was considered a success both by health authorities and by local communities and their leaders, and was, as a result of the positive feedback, expanded to all six study LGAs in 2010. A larger scale-up of the CDI programme to seven states supported to the World Bank Booster Programme is currently under review by the Nigerian National Malaria Control Programme (NMCP) and the World Bank.

## Competing interests

The authors declare that they have no competing interests.

## Authors' contributions

JCO led data collection at both baseline and endline. He was responsible for overseeing data quality, cleaning, entry and preliminary analysis. He also provided technical assistance for training and field supervision, and contributed in drafting the manuscript. BCO served as project manager. He guided all aspects of project implementation in the field including training, supervision, and advocacy with local health authorities, and assurance of fidelity of intervention across all clinics and communities. WB was responsible for project design based on a preliminary situation analysis that he co-led. He oversaw development of data collection instruments and training materials. As principal investigator he provided overall project supervision and guidance. GI assisted in instrument development and data management with particular attention of all aspects of monitoring and evaluation and clinic quality performance.

EO co-led the initial situation analysis and offered regular technical assistance on the clinic performance standard improvement component of the project based on national guidelines that he helped develop. BR assisted in instrument development and provided technical assistance for monitoring and evaluation during the project. EUN served as assistant project manager responsible for all logistics and field supervision of intervention including monthly supervisory and problem solving meetings with community volunteers and health staff. NO assisted with data management, preliminary analysis and production of the study report that formed the basis of the initial drafting of the manuscript. GF guided final data analysis and coordinated the whole team in the writing of the manuscript. All authors read and approved the final manuscript.
